# Circulating prostasin is an independent marker of mortality risk in patients with idiopathic pulmonary fibrosis

**DOI:** 10.1183/23120541.00738-2024

**Published:** 2025-06-23

**Authors:** Jamie L. Todd, Courtney Page, Peitao Wu, John A. Belperio, Toby M. Maher, Scott M. Palmer, Thomas B. Leonard, Christian Hesslinger, Megan L. Neely, Thomas Schlange

**Affiliations:** 1 Duke Clinical Research Institute, Durham, NC, USA; 2 Duke University Medical Center, Durham, NC, USA; 3 Boehringer Ingelheim Pharmaceuticals, Inc., Ridgefield, CT, USA; 4 David Geffen School of Medicine at UCLA, Los Angeles, CA, USA; 5 Keck School of Medicine, University of Southern California, Los Angeles, CA, USA; 6 National Heart and Lung Institute, Imperial College London, London, UK; 7 Boehringer Ingelheim Pharma GmbH & Co. KG, Biberach, Germany; 8 These authors contributed equally to this work

## Abstract

**Background:**

Prostasin is expressed in the lung epithelium where it regulates fluid and electrolyte balance *via* sodium channel proteolysis. We investigated whether circulating prostasin levels are associated with the presence and severity of idiopathic pulmonary fibrosis (IPF) and whether prostasin levels, or changes in them, are associated with mortality.

**Methods:**

Patients with IPF came from the IPF-PRO Registry. Controls without lung disease had a similar age/sex distribution. Prostasin was quantified in plasma taken at enrolment and, in the IPF cohort, ∼6 months post-enrolment, by immunoassay. Linear regression was used to compare prostasin levels at enrolment in patients with IPF *versus* controls and, in the IPF cohort, determine associations between prostasin level and lung function. Multivariable Cox proportional hazards models determined associations between prostasin level at enrolment and change in prostasin level over 6 months and respiratory death.

**Results:**

Prostasin level at enrolment was higher in patients with IPF (n=624) *versus* controls (n=100) (fold-difference 1.75; p<0.001). In the IPF cohort, the difference in disease severity per 1 standard deviation (sd) difference in prostasin was −3.85 for forced vital capacity % predicted and −4.24 for diffusing capacity of the lung for carbon monoxide % predicted (both p<0.001). The adjusted hazard ratio (HR) for respiratory death per 1 sd difference in prostasin at enrolment was 1.20 (95% CI 1.04–1.40, p=0.014, n=624). The adjusted HR for subsequent respiratory death per 1 sd difference in change in prostasin over 6 months was 1.33 (95% CI 1.01–1.74, p=0.041, n=290).

**Conclusions:**

Circulating prostasin is an independent marker of mortality risk in patients with IPF.

## Shareable abstract

**In patients with idiopathic pulmonary fibrosis in the IPF-PRO Registry, the circulating level of prostasin was an independent marker of mortality risk** https://bit.ly/3D0Vmhx

## Introduction

Idiopathic pulmonary fibrosis (IPF) is a progressive fibrosing interstitial lung disease (ILD) associated with decline in lung function and high mortality [1]. IPF has a variable clinical course and there remains an unmet need to identify prognostic biomarkers. The pathobiology of IPF is believed to involve aberrant responses in airway and alveolar epithelial cells. These cells may serve as sensors, integrating the complex interplay of genetic and environmental risk factors, processes associated with ageing and profibrotic responses to injury [2, 3]. Epithelial-derived protein biomarkers have shown promise with regard to risk stratification in patients with IPF [4–7].

Prostasin is a trypsin-like serine protease expressed in epithelial cells where it regulates fluid and electrolyte balance *via* sodium channel proteolysis [8] and modulates signalling mediated by the epidermal growth factor receptor [9]. In a prospective study, a higher level of circulating prostasin was associated with the presence of IPF, but not with progression of IPF over 12 months [10]. In a study of 385 patients with progressive fibrosing ILDs other than IPF, prostasin was selected as part of a multiprotein signature that predicted 12-month progression [11]. In a recent analysis of data from 871 patients with IPF in the Pulmonary Fibrosis Foundation patient registry, prostasin was among the proteins most strongly associated with 3-year transplant-free survival [12]. While these studies have identified prostasin as a potential prognostic biomarker in patients with ILDs, they have largely relied on semi-quantitative data generated by proximity extension assays. Weak correlations have been observed between some proteins measured by such methods compared to quantitative assays [12], suggesting that they have poor specificity. In addition, it remains uncertain whether longitudinal measurement of prostasin could add prognostic information over a single measurement, or whether the use of antifibrotic therapies influences prostasin levels or their prognostic significance.

The IPF-PRO Registry is a multicentre US registry of patients with IPF [13]. We used data from this registry to investigate associations between prostasin levels and the presence and severity of IPF and between prostasin levels at enrolment and during early follow-up and mortality.

## Materials and methods

### Study population

Patients with IPF that was diagnosed or confirmed at the enrolling centre in the past 6 months were enrolled into the IPF-PRO Registry (NCT01915511) at 46 sites across the USA. Data for this analysis came from 624 patients with prostasin measurements at enrolment, of whom 292 (46.8%) also had a prostasin measurement at 6±3 months post-enrolment, with most samples collected within 1 month of the 6-month timepoint (supplementary figure S1). A control cohort was drawn from the Measurement to Understand the Reclassification of Disease of Cabarrus/Kannapolis (MURDOCK) study, a longitudinal cohort study of adults in North Carolina in which self-reported health information and biological samples are collected [14]. The control cohort comprised 100 individuals of similar age, sex and smoking status distribution to the IPF cohort without known lung disease (see supplementary material).

The IPF-PRO Registry obtained ethics approval at the data coordinating centre (Duke Clinical Research Institute, Duke Institutional Review Board Protocol Number Pro00046131) and at every enrolling centre (listed in the Acknowledgements). Ethics approval was granted by the Duke Institutional Review Board Protocol Number Pro00082241 to use the biosamples obtained as part of the IPF-PRO Registry for the analyses contained herein. All participants provided written informed consent. The MURDOCK study community registry and biorepository was approved by the Duke University Health Institutional Review Board (Pro00011196), and all participants provided written informed consent.

### Prostasin quantification

Prostasin levels were quantified in plasma samples using immunoassay (Myriad RBM, Austin, TX, USA). All samples rendered values (µg·L^−1^) within the range of assay detection.

### Statistical analysis

Descriptive statistics were used to analyse baseline characteristics in the IPF and control cohorts. Linear regression was used to compare prostasin levels between the IPF cohort (overall and by use of antifibrotic therapy (nintedanib or pirfenidone) at enrolment) and the control cohort. Scatter plots and Spearman's correlation coefficients (rho) were used to describe relationships between prostasin level and continuous measures of disease severity at enrolment including forced vital capacity (FVC) and diffusing capacity of the lung for carbon monoxide (*D*_LCO_) % predicted. Linear regression was used to determine associations between prostasin level and continuous measures of disease severity, reported as the difference in disease severity per 1 standard deviation (sd) difference in prostasin level. These analyses were performed in the overall cohort unadjusted and adjusted for use of antifibrotic therapy at enrolment.

The cumulative incidence of respiratory death stratified by prostasin level above or below the median at enrolment was described in the overall IPF cohort and in subsets by use of antifibrotic therapy at enrolment. Cox proportional hazards models, unadjusted and adjusted for age, sex, FVC % predicted and *D*_LCO_ % predicted at enrolment, were used to determine the association between prostasin level at enrolment and respiratory death in the overall IPF cohort. The area under the receiver operating curve (ROC) was computed at 12 and 24 months after enrolment to assess the discriminative ability of the adjusted model for respiratory death.

The absolute change in prostasin between enrolment and 6 months was described in the overall IPF cohort and in subsets defined by the pattern of antifibrotic therapy (continued antifibrotic therapy, initiated antifibrotic therapy, or remained untreated) over the same period. Correlations between the absolute change in prostasin over 0–6 months and absolute changes in FVC % predicted and *D*_LCO_ % predicted over 0–6 months and 0–12 months were assessed using the Spearman correlation coefficient. Associations between absolute change in prostasin over 0–6 months and subsequent respiratory death were analysed using Cox proportional hazards models landmarked at the follow-up sample collection date. In a minimally adjusted model, the model was adjusted for the enrolment prostasin level only. In a fully adjusted model, the model was adjusted for prostasin, age, sex, FVC % predicted and *D*_LCO_ % predicted, all assessed at enrolment. The area under the ROC was computed at 12 and 24 months after the follow-up sample to assess the discriminative ability of the adjusted model for respiratory death.

To understand internal validity, for the baseline and longitudinal risk modelling two-step iterative resampling was used to test the internal validity of the associations. The analysis cohort was randomly split into discovery and replication cohorts in a 7:3 ratio and 100 random splits were taken. The model was considered validated if p1<α1=0.1 and p2<α2=0.5 in split1 and split2, respectively [15]. We assessed the proportion of 100 random splits where the association was validated. Findings were to be considered internally robust if they were replicated in ≥20% of the random splits [15].

As prostasin is known to be expressed in male tissues (*i.e.* testes, prostate) [16], for all models, interactions between prostasin and sex were examined. In the event of a significant interaction (interaction p-value <0.05), results were presented overall and stratified by sex.

Multiple imputation was used to account for missing data for adjustment covariates and missing data were filled in five times to generate five complete data sets as per the Full Conditional Specification method. The five complete data sets were analysed using the models described above, and the results from the five complete datasets were combined using Rubin's rules to produce the final inferential results [17].

## Results

### Cohort characteristics

At enrolment, the IPF cohort (n=624) had a mean±sd age of 69.8±7.8 years; 74.4% were male, 91.1% were white and 66.8% had a history of smoking (table 1). Mean±sd FVC % predicted and *D*_LCO_ % predicted were 72.5±18.5 and 43.6±15.1, respectively. Almost half (48.4%) of the cohort were taking antifibrotic therapy (table 1). In the control cohort (n=100), the mean±sd age was 66.7±5.2 years, 74.0% were male, all subjects were white, and 68.0% had a history of smoking.

**TABLE 1:** Characteristics of the idiopathic pulmonary fibrosis cohort at enrolment (n=624)

**Age years**	69.8±7.8
**Male**	464 (74.4)
**White^#^**	500 (91.1)
**Ever-smoker** ^¶^	416 (66.8)
**FVC % predicted**	72.5±18.5
***D***_**LCO**_ **% predicted**	43.6±15.1
**Taking antifibrotic therapy**	302 (48.4)
Nintedanib	157 (25.2)
Pirfenidone	145 (23.2)

Data are presented as n (%) or mean±sd. FVC: forced vital capacity; *D*_LCO_: diffusing capacity of the lung for carbon monoxide. ^#^: n=549 analysed. ^¶^: n=623 analysed.

### Prostasin level and IPF presence/severity

**FIGURE 1:**
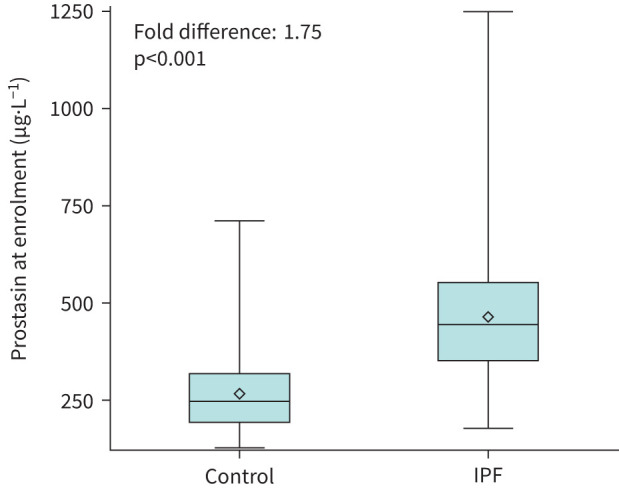
Prostasin levels at enrolment in the control and idiopathic pulmonary fibrosis (IPF) cohorts. Markers in the boxes denote means, midlines of the boxes medians, boundaries of the boxes 25th and 75th percentiles, whiskers minimum and maximum values.

At enrolment, the mean±sd prostasin level was 466.3±158.8 µg·L^−1^ in the IPF cohort and 267.0±107.6 µg·L^−1^ in the control cohort (figure 1). The prostasin level was significantly higher in patients with IPF *versus* controls (fold-difference 1.75; p<0.001), a finding that was similar in both females and males (fold-differences of 2.08, p<0.001 and 1.66, p<0.001, respectively). The prostasin level was higher in patients with IPF *versus* controls regardless of use of antifibrotic therapy (supplementary table S1).

**FIGURE 2:**
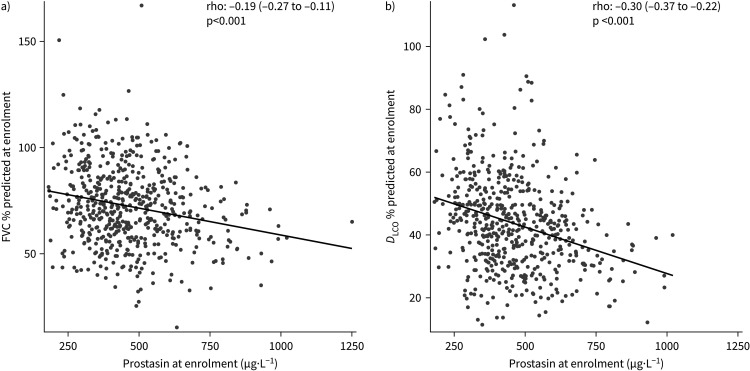
Scatter plots of prostasin level and a) forced vital capacity (FVC) % predicted or b) diffusing capacity of the lung for carbon monoxide (*D*_LCO_) % predicted at enrolment. Spearman correlation coefficients with 95% confidence intervals are shown and the fitted linear regression model is overlaid.

In the IPF cohort, prostasin levels were higher among patients with lower FVC or *D*_LCO_ % predicted at enrolment (figure 2). The difference in disease severity per 1 sd difference in prostasin level was −3.85 for FVC % predicted and −4.24 for *D*_LCO_ % predicted (both p<0.001). These associations were similar after adjustment for use of antifibrotic therapy (−3.85 for FVC % predicted and −4.28 for *D*_LCO_ % predicted). There was a significant interaction between enrolment prostasin level and sex with respect to FVC % predicted (interaction p=0.002), with a difference in FVC % predicted per 1 sd difference in prostasin level of −3.22 in females and −4.10 in males. There was no significant interaction between enrolment prostasin and sex with respect to *D*_LCO_ % predicted (interaction p=0.129).

### Prostasin level and respiratory death

**FIGURE 3:**
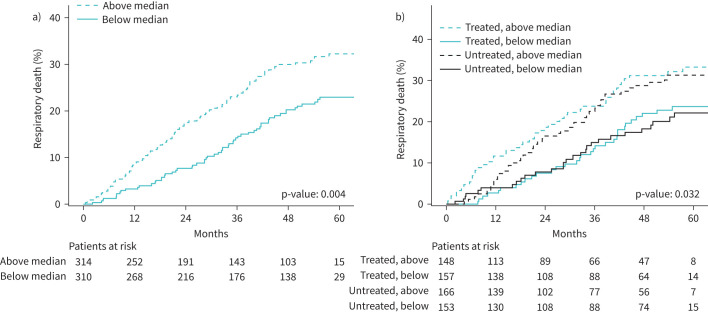
Cumulative incidence of respiratory death over follow-up a) stratified by prostasin level above or below the median or b) stratified by prostasin level and antifibrotic therapy at enrolment.

Over a median follow-up of 37.2 months, the cumulative incidence of respiratory death was higher among patients with a prostasin level above *versus* below the median at enrolment (figure 3a). This finding was consistent between patients taking *versus* not taking antifibrotic therapy at enrolment (figure 3b). Among all patients, enrolment prostasin level was significantly associated with a higher risk for respiratory death (adjusted hazard ratio (HR) per 1 sd difference in prostasin level 1.20; 95% CI 1.04–1.40; p=0.014) (supplementary figure S2). Two-step iterative resampling demonstrated the robustness of these findings, with the association validated in 97% and 47% of the random splits for the unadjusted and adjusted analyses, respectively. The adjusted model including enrolment prostasin level had good discriminatory ability with respect to the risk of respiratory death at 12 or 24 months post-enrolment (supplementary figure S3).

**FIGURE 4:**
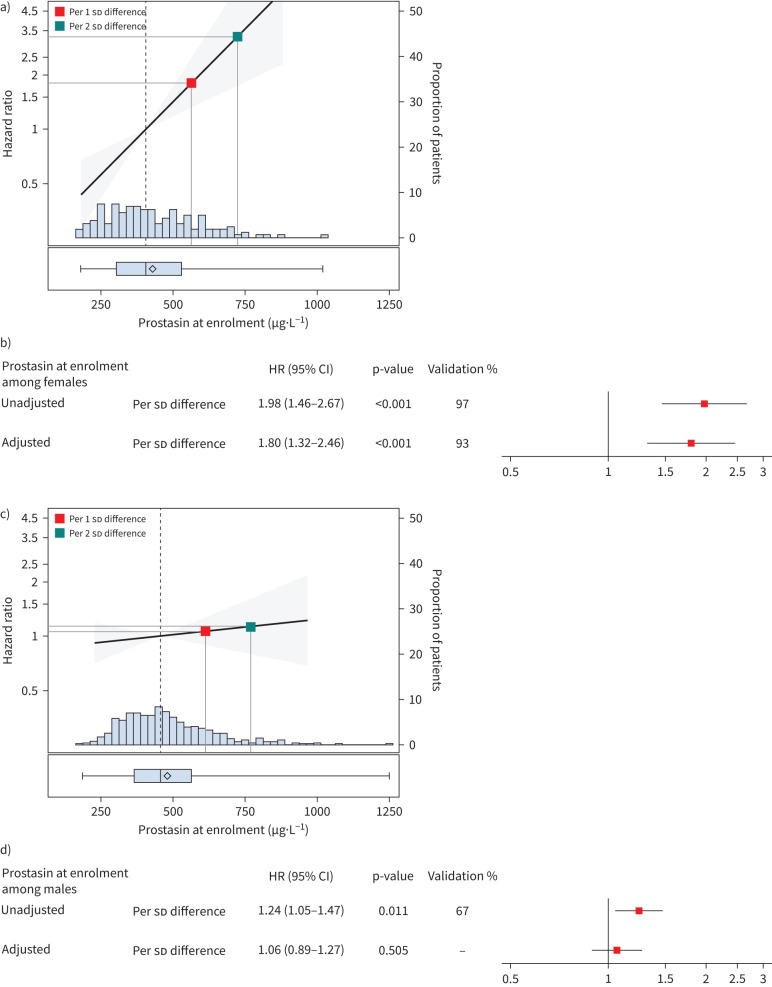
Analyses of enrolment prostasin level and the outcome of respiratory death, stratified by sex. a) Among females, distribution of prostasin levels at enrolment and estimated hazard ratios (HRs) and 95% CIs for respiratory death based on an adjusted Cox model. The red square is the estimated HR per 1 sd difference in prostasin level at enrolment. The green square is the estimated HR per 2 sd difference in prostasin level at enrolment. b) Among females, estimated associations between prostasin levels at enrolment and time to respiratory death in unadjusted and adjusted Cox models. c) Among males, distribution of prostasin levels at enrolment and estimated HRs and 95% CIs for respiratory death based on an adjusted Cox model. The red square is the estimated HR per 1 sd difference in prostasin level at enrolment. The green square is the estimated HR per 2 sd difference in prostasin level at enrolment. d) Among males, estimated associations between prostasin levels at enrolment and time to respiratory death in unadjusted and adjusted Cox models.

**FIGURE 5:**
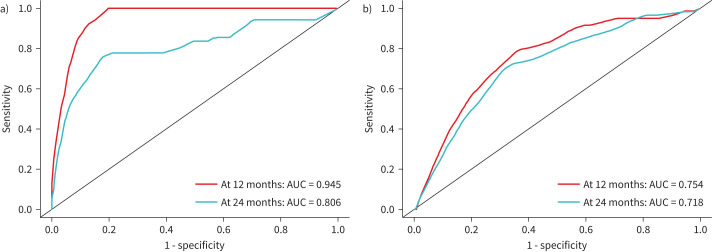
Receiver operating curve for the adjusted model including enrolment prostasin level for respiratory death at 12 months or 24 months after enrolment, stratified by sex: a) females; b) males. AUC: area under the curve.

Interaction analyses demonstrated a significant interaction between enrolment prostasin level and sex with respect to the outcome of respiratory death (interaction p=0.006). As shown in figure 4, prostasin levels at enrolment were more strongly associated with respiratory death among females (unadjusted HR 1.98; 95% CI 1.46–2.67; p<0.001; validated in 97% of the random splits) than among males (unadjusted HR 1.24; 95% CI 1.05–1.47; p=0.011; validated in 67% of the random splits). After adjustment for age, sex, FVC % predicted and *D*_LCO_ % predicted at enrolment, the significant association of prostasin with respiratory death persisted among females (adjusted HR 1.80; 95% CI 1.32–2.46; p<0.001; validated in 93% of the random splits) but was attenuated among males (adjusted HR 1.06; 95% CI 0.89–1.27; p=0.505). The adjusted model including enrolment prostasin level demonstrated excellent ability to discriminate respiratory death at 12 or 24 months post-enrolment among females (C-indices of 0.95 and 0.81, respectively), but the performance of the model was less robust for males (C indices of 0.75 and 0.72, respectively) (figure 5).

### Changes in prostasin levels and outcomes

In the subset of patients with prostasin measurements 6±3 months post-enrolment (n=292), 45.9% of patients experienced an increase in prostasin level, 53.4% a decrease in prostasin level and 0.7% no change in prostasin level. Absolute changes in prostasin level over 6 months were generally similar across groups based on pattern of antifibrotic therapy use (continued antifibrotic therapy, initiated antifibrotic therapy, remained untreated) (supplementary table S2).

**FIGURE 6:**
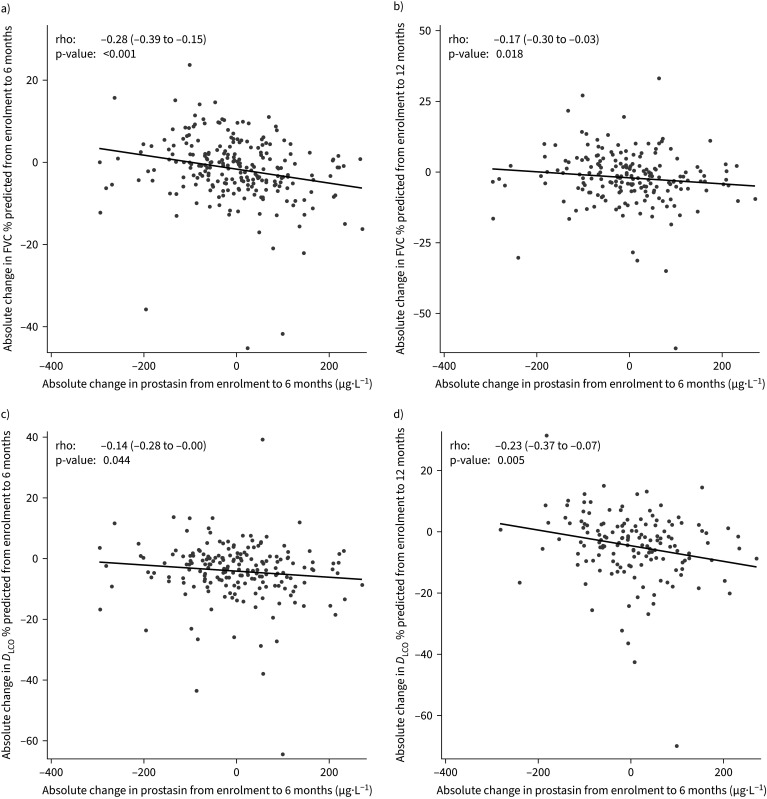
Relationship between absolute change in prostasin from enrolment to 6 months and a) absolute change in forced vital capacity (FVC) % predicted from enrolment to 6 months, b) absolute change in FVC % predicted from enrolment to 12 months, c) absolute change in diffusing capacity of the lung for carbon monoxide (*D*_LCO_) % predicted from enrolment to 6 months, and d) absolute change in *D*_LCO_ % predicted from enrolment to 12 months.

Absolute changes in prostasin from enrolment to 6 months were significantly correlated with lung function changes over this same time-frame (figure 6). Specifically, there was a moderate negative correlation between absolute change in prostasin level over 0–6 months and absolute change in FVC % predicted over the same period (rho= −0.28; 95% CI −0.39 to −0.15). The correlation between absolute change in prostasin level over 0–6 months and absolute change in FVC % predicted over 0–12 months was also moderately negative (rho= −0.17; 95% CI, −0.30 to −0.03) (figures 6a and b). There were weak to moderate negative correlations between absolute change in prostasin level over 0–6 months and absolute change in *D*_LCO_ % predicted over 0–6 months (rho= −0.14; 95% CI −0.28 to 0.00) or 0–12 months (rho= −0.23; 95% CI −0.37 to −0.07) (figures 6c and d).

**FIGURE 7:**
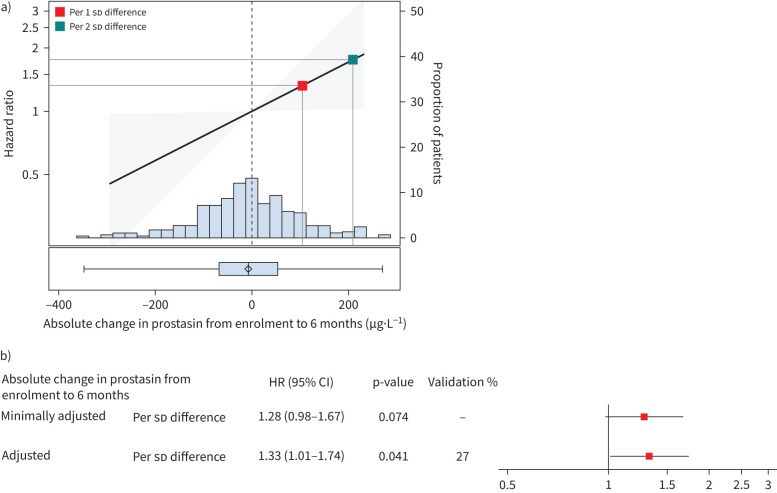
Analyses of absolute change in prostasin in patients with idiopathic pulmonary fibrosis (IPF). a) Distribution of absolute change in prostasin over 6 months post-enrolment and estimated hazard ratios (HRs) and 95% CIs for respiratory death from adjusted Cox model landmarked at the 6-month blood collection date. The red square is the estimated HR per 1 sd difference in absolute change in prostasin level over 6 months. The green square is the estimated HR per 2 sd difference in the absolute change in prostasin level over 6 months. b) Estimated associations between absolute change in prostasin over 6 months and respiratory death based on minimally and fully adjusted models.

**FIGURE 8:**
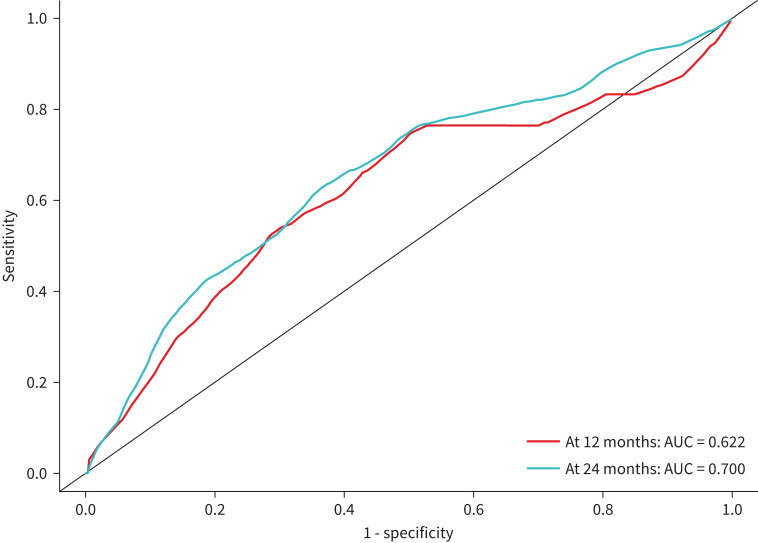
Receiver operating curve for the adjusted model including absolute change in prostasin level between enrolment and 6 months for respiratory death at 12 months or 24 months after the follow-up blood sample. AUC: area under the curve.

The results of the Cox models of associations between absolute change in prostasin level over 0–6 months and subsequent respiratory death are shown in figure 7. In minimally adjusted models, the HR per 1 sd difference in change in prostasin level over 6 months was 1.28 (95% CI 0.98 to 1.67; p=0.074). In the fully adjusted model, the HR per 1 sd difference in change in prostasin level over 6 months was 1.33 (95% CI 1.01 to 1.74; p=0.041). The HR per 1 sd difference was validated in 27% of the random splits. There was no evidence of a significant interaction between change in prostasin over 6 months and sex (interaction p=0.521), suggesting that the influence of short-term changes in circulating prostasin on the risk of respiratory death was similar in females and males. The adjusted model for respiratory death 12 or 24 months after the follow-up blood sample that included absolute change in prostasin over 6 months showed good discriminatory ability (figure 8).

## Discussion

In this study, we demonstrated that circulating levels of prostasin were associated with the presence of IPF and were highly correlated with measures of the severity of IPF, irrespective of use of antifibrotic therapy. The associations between prostasin level and measures of lung function were stronger than those observed for other epithelial-derived protein biomarkers studied in the IPF-PRO Registry cohort, including surfactant protein D [18]. Higher levels of prostasin at enrolment were associated with an increased risk of respiratory death during follow-up. This finding was particularly striking among females, in whom the effect persisted after adjustment for clinical variables that may influence mortality. We also demonstrated an association between change in prostasin level over 6 months and subsequent risk of respiratory death, suggesting that dynamic changes in prostasin level may provide information about mortality risk beyond that provided by a single measurement. To our knowledge, this is the first report on the prognostic potential of prostasin in a contemporary cohort of patients with IPF based on quantitative measurements.

Evidence of the potential role of prostasin in pulmonary disease comes mainly from research in cystic fibrosis. The expression of processed prostasin is increased on the surface of airway epithelial cells from patients with cystic fibrosis [19], and in these cells, inhibition of prostasin and other channel-activating proteases improves mucociliary function [20]. Among patients with cystic fibrosis, sputum trypsin-like protease activity was inversely correlated with FVC % predicted and was higher in individuals who died within the following 5 years [21]. Nasal administration of a prostasin inhibitor reduced sodium transport in the airway of patients with cystic fibrosis [22]. While these data support the idea that prostasin dysregulation may play a role in the progression of lung disease, the mechanisms of its involvement in the pathobiology of IPF are yet to be determined.

In the search for prognostic biomarkers in IPF, the potential impact of antifibrotic therapy should be considered. In a recent analysis of data from 48 patients with IPF, among 26 proteins studied, seven, including surfactant proteins A1 and D and intercellular adhesion molecule 1, were elevated in patients receiving *versus* not receiving antifibrotic therapy, but associations with outcomes were not assessed [23]. In a study conducted in 325 patients with IPF, several protein biomarkers associated with prognosis in the era before the availability of antifibrotic therapy were shown to predict mortality both in patients who were and were not receiving antifibrotic therapy, but the thresholds predictive of reduced survival were higher in the patients receiving antifibrotic therapy [24]. In our analyses, we found that a higher prostasin level was associated with the presence of IPF and with worse lung function in patients with IPF irrespective of use of antifibrotic therapy. Further, our finding that the incidence of respiratory death was higher among patients with a higher prostasin level at enrolment was consistent between patients taking *versus* not taking antifibrotic therapy at enrolment.

Our findings highlight the importance of considering sex as a variable in the search for prognostic biomarkers in IPF. This may be particularly relevant when considering circulating biomarkers. Our analyses demonstrated a much stronger association between enrolment prostasin and respiratory death in females than males. This association persisted in females after accounting for clinical factors that may influence outcomes such as lung function. A model that included enrolment prostasin level had excellent discriminatory ability for respiratory death in females, while model performance metrics were more modest in males.

Strengths of our analyses include the quantitative measurement of prostasin, the longitudinal assessment of prostasin levels that enabled us to analyse associations between changes in prostasin levels and respiratory death, and the assessment of associations in subgroups based on use of antifibrotic therapy. Our internal validation approach supported the robustness of our observations. Our study also had limitations. Although we employed a quantitative assay for prostasin measurement, the assay employed is not a clinical grade assay and further assay validation should be conducted. While our findings suggest that prostasin is a promising biomarker of disease progression in patients with IPF, we acknowledge that a multiprotein signature will likely be needed to provide an accurate assessment of prognosis in these patients. Finally, while our analyses adjusted for several clinical variables that may confound the relationship between prostasin level and outcomes in patients with IPF, there could be other unmeasured confounders.

### Conclusions

In a real-world prospective multicentre cohort of patients with IPF, circulating levels of prostasin were highly correlated with measures of disease severity. Further, the prostasin level at baseline, and the absolute change in prostasin level over 6 months, were independently associated with risk of mortality. These findings support the value of prostasin as a prognostic biomarker in patients with IPF.

## Data Availability

The datasets analysed during the current study are not publicly available but are available from the corresponding author on reasonable request.

## Supplementary material

**Please note:** supplementary material is not edited by the Editorial Office, and is uploaded as it has been supplied by the author. Supplementary material 00738-2024.SUPPLEMENT
