# Supplementary material for: Circulating prostasin is an independent marker of mortality risk in patients with idiopathic pulmonary fibrosis
Source: ERJ Open Res. 2025 Jun 23;11(3):00738-2024. doi: 10.1183/23120541.00738-2024 (PMC12183712; doi:10.1183/23120541.00738-2024)

## Supplemental Material

## Control Cohort

Controls were drawn from the Measurement to Understand the Reclassification of Disease of Cabarrus/Kannapolis (MURDOCK) Study, a cohort study of adults in North Carolina. Participants considered for inclusion as controls were white and non-Hispanic, aged 60 to 80 years, with an enrolment blood sample. Participants were excluded if they had self-reported respiratory disease, cancer, or autoimmune disease, were active smokers, had second-hand tobacco exposure, or reported use of respiratory-targeted medications or immunomodulators. Stratified random sampling (stratification on sex and smoking status [ever/never]) was used to select 100 controls.

**Table S1.** Association between prostasin level and the presence of IPF in the overall cohort and stratified by antifibrotic therapy at enrollment.

| | Fold difference vs controls |
|---|---|
| All patients with IPF (n=624*) | 1.75 |
| Taking nintedanib (n=157) | 1.66 |
| Taking pirfenidone (n=145) | 1.79 |
| Not taking antifibrotic therapy (n=319) | 1.77 |

*Three patients were recorded as being on both nintedanib and pirfenidone at enrollment and were excluded from the treatment subset analyses.

**Table S2.** Absolute change in prostasin from enrollment to 6 months in the overall IPF cohort and based on the pattern of antifibrotic therapy use over the same period.

| | **Patients with absolute decrease in prostasin** | | | **Patients with absolute increase in prostasin** | | |
|---|---|---|---|---|---|---|
| | **N** | **Mean ± SD** | **Min, Max** | **N** | **Mean ± SD** | **Min, Max** |
| Overall[a] | 156 | -82.3 ± 71.0 | -348, -2.0 | 134 | 78.6 ± 65.4 | 1.0, 271.0 |
| Continued treatment | 73 | -82.9 ± 78.3 | -348, -4.0 | 69 | 70.9 ± 59.5 | 1.0, 231.0 |
| Discontinued treatment | 1 | -82.0 | -82.0, -82.0 | 3 | 56.7 ± 62.6 | 7.0, 127.0 |
| Initiated treatment | 43 | -87.9 ± 65.6 | -240, -2.0 | 35 | 77.1 ± 68.9 | 1.0, 271.0 |
| No treatment | 39 | -74.8 ± 64.1 | -269, -6.0 | 27 | 102.4 ± 72.8 | 5.0, 266.0 |

[a]Two patients had no change in prostasin level between enrollment and 6 months and were excluded from this table.

**Figure S1.** Distribution of time from enrollment to follow-up sample collection (n=292).

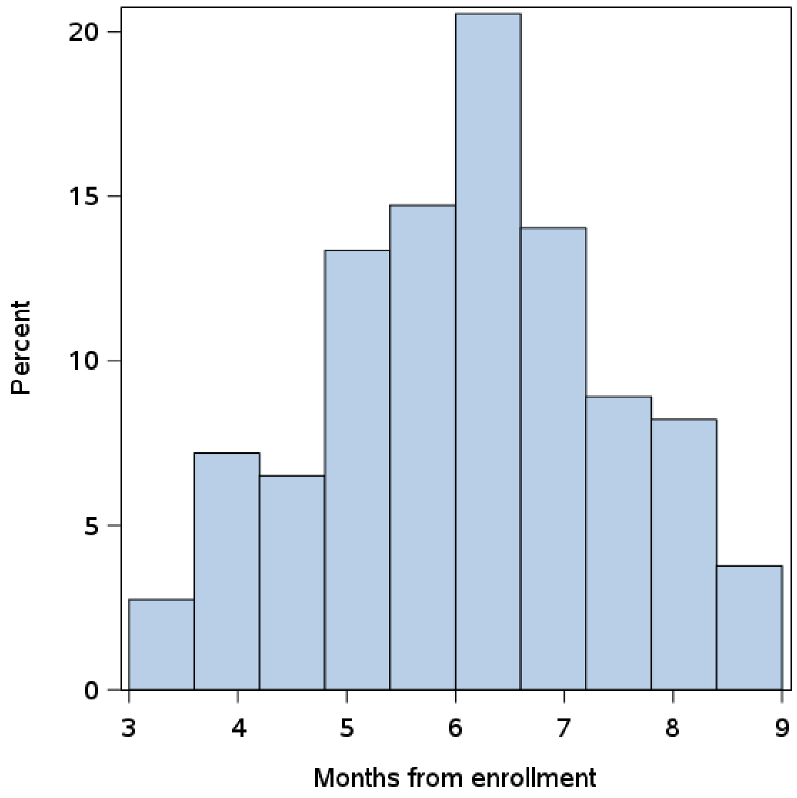

**Figure S2.** Analyses of enrollment prostasin level and the outcome of respiratory death in patients with IPF. (**A**) Distribution of prostasin levels at enrollment and estimated HRs and 95% CIs for respiratory death based on an adjusted Cox model. The red square is the estimated HR per 1 SD difference in prostasin level at enrollment. The green square is the estimated HR per 2 SD difference in prostasin level at enrollment. (**B**) Estimated associations between prostasin levels at enrollment and time to respiratory death in unadjusted and adjusted Cox models.

**A**

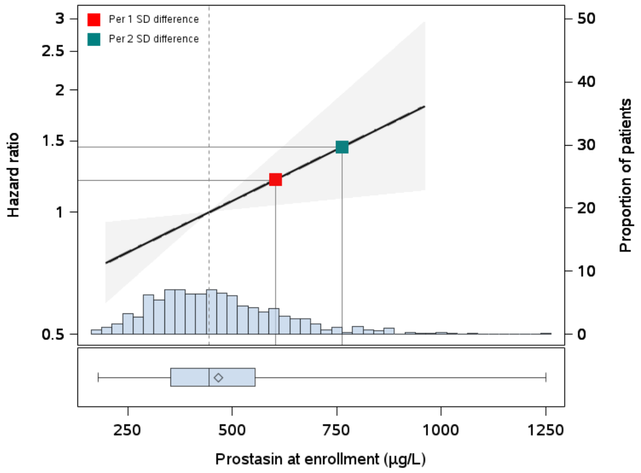


**B**

| Prostasin at enrollment | | HR (95% CI) | P-value | Validation % |
|---|---|---|---|---|
| Unadjusted | Per SD difference | 1.37 (1.19 - 1.57) | <.001 | 97% |
| Adjusted | Per SD difference | 1.20 (1.04 - 1.40) | 0.014 | 47% |

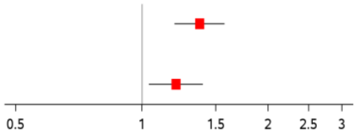

**Figure S3.** Receiver operating curve for the adjusted model including enrollment prostasin level for respiratory death at 12 months (red line) or 24 months (blue line) after enrollment.

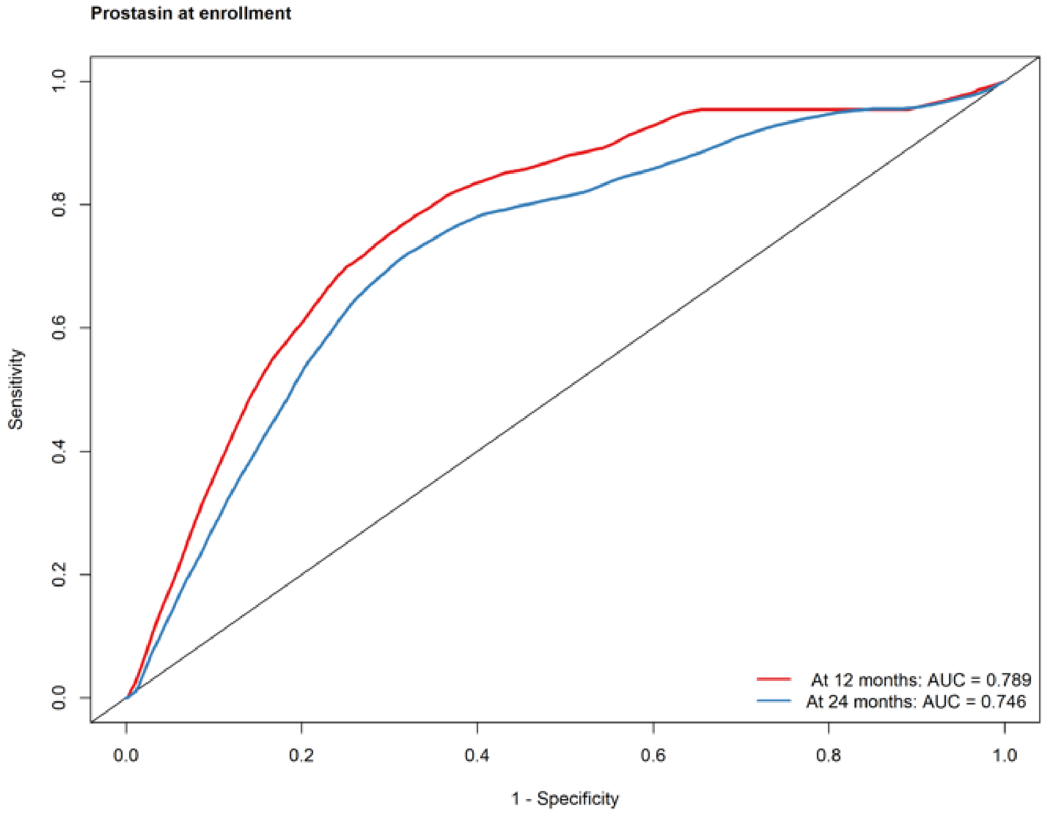

Supplement: Supplementary file 1 [file 00738-2024.SUPPLEMENT.pdf]
